# Genome wide association study of vaginal microbiota genetic diversity in French women

**DOI:** 10.12688/openreseurope.20462.2

**Published:** 2026-03-11

**Authors:** Samuel Alizon, Claire Bernat, Vanina Boué, Sophie Grasset, Soraya Groc, Tsukushi Kamiya, Massilva Rahmoun, Christian Selinger, Nicolas Tessandier, Marine Bonneau, Vincent Foulongne, Christelle Graf, Jacques Reynes, Michel Segondy, Vincent Tribout, Jacques Ravel, Nathalie Boulle, Carmen Lia Murall, Vincent Pedergnana, Jean-François Deleuze

**Affiliations:** 1 CIRB, INSERM, Collège de France, Université PSL, Centre National de la Recherche Scientifique (CNRS), Paris, Île-de-France, France; 2 MIVEGEC, IRD, Université de Montpellier, Centre National de la Recherche Scientifique, Montpellier, Occitanie, France; 3 Institut de Génomique Fonctionnelle, Universite de Montpellier, Montpellier, Occitanie, France; 4 PCCEI, Inserm, University Montpellier, Montpellier, Occitanie, France; 5 Swiss Tropical and Public Health Institute, Basel, Switzerland; 6 Department of Obstetrics and Gynaecology, Centre Hospitalier Universitaire de Montpellier, Montpellier, France; 7 Department of Infectious and Tropical Diseases, Centre Hospitalier Universitaire de Montpellier, Montpellier, France; 8 Institute for Genome Sciences, Department of Microbiology and Immunology, University of Maryland School of Medicine, Baltimore, Maryland, USA; 9 National Microbiology Laboratory (NML), Montreal Public Health Agency of Canada (PHAC), Winnipeg, Canada; 10 Centre National de Recherche en Génomique Humaine (CNRGH),, Université Paris-Saclay, Évry-Courcouronnes, France

**Keywords:** vaginal microbiota, human genetics, GWAS, SNP, variants, diversity, CST

## Abstract

**Background:**

The composition of the vaginal microbiota is known to be highly structured into five main community state types (CSTs) that are found in all human populations. Several associations between perceived ethnicity and the type of community have been reported but analyses of human genetic data, especially genome wide association studies (GWAS), remain limited and mostly rely on phenotypic traits rather than microbial DNA data.

**Methods:**

Analysing genotyping data from in 168 women from the PAPCLEAR cohort study in France, we perform a GWAS looking for human genetic polymorphisms associated with vaginal microbiota community composition. For the latter, we use Simpson diversity and community state type (CST) as summary statistics to summarise 16S RNA metabarcoding data.

**Results:**

We show that inverse Simpson diversity is the trait related to the vaginal microbiota that is best explained by the human genome. Furthermore, we identify several genomic regions associated with variations in this trait and show that the covariates associated with vaginal microbiota composition do not correlate with these genetic variants.

**Conclusion:**

This is one of the first GWAS to use microbial genetic data instead of symptoms to characterise the vaginal microbiota. However, it remains limited because of the size of our cohort and our results call for more powered studies in terms of participants and genome coverage.

## Plain Language Summary

In all the populations sampled across the world, the composition of the vaginal microbiota falls into five main community state types (CST), four of which are dominated by a single species of Lactobacillus bacteria. Furthermore, the relative frequency of these CSTs consistently varies according to covariates related to age or lifestyle. In particular, several studies report that CST frequency vary with self-reported ethnicity. However, to date, very few genome wide association studies (GWAS) have been performed on the diversity of the vaginal microbiota to identify genetic variants that could explain the observed variation.

Building on the PAPCLEAR cohort, which was set up in Montpellier (France) to study HPV infections and the vaginal microbiota, we perform a GWAS using the Shannon diversity of the vaginal microbiota as our response trait. We identify two genomic regions associated with microbiota diversity variations.

This is one of the first GWAS to use microbial genetic data instead of symptoms to characterise the vaginal microbiota and our results call for more powered studies in terms of participants and genome coverage.

## Introduction

The vaginal microbiota has been studied since the end of the 19th century, which is consistent with the massive impact it is now known to have on women’s susceptibility to sexually-transmitted infections, ^1^ fertility, ^2^ and general well-being. ^3^ The vaginal microbiota is also highly structured into five main community state types, or CSTs, ^4^ which tend to be relatively stable over several months. ^5^^,^^6^ Its composition has also been long reported to be associated with perceived ethnicity. ^4^^,^^7^^–^^9^

Contrarily to other human microbiota, ^10^ only a few studies, which are detailed below, have attempted to identify potential genetic factors associated with the observed variation in human vaginal microbiota. This is surprising since all studies conducted around the globe detect the same five main CSTs. Four of these communities are dominated, sometimes exclusively, by a single species of *Lactobacillus* ( *L. crispatus* for CST-1, *L. jenseni* for CST-2, *Lactobacillus iners* for CST-3, and *Lactobacillus gasseri* for CST-5). Conversely, the fifth one, CST-4, is a diverse assemblage of anaerobic bacteria from genera such as *Gardnerella*, *Prevotella*, or *Fannyhessea.* It is also often associated with bacterial vaginosis (BV) and other health issues. ^24^^,^^4^^,^^1^^,^^28^ The relative proportion of each CST can vary widely between populations, although CST-1, CST-3, and CST-4 are always the most frequent. In North America, CST-4 was found to be four times more common in women who identify themselves as ‘Black’ or ‘Hispanic’ than in those who identify themselves as ‘White’. ^4^ These differences could have behavioural origins. For example, vaginal douching is known to be strongly associated with CST-4 and this practice varies across populations. ^15^ However, to date, we do not know to what extent this difference is associated with human genetics. One of the motivations to identify potential single nucleotide polymorphisms (SNPs) associated with CST-4, also sometimes referred to as ‘molecular bacterial vaginosis’, ^16^ is that half of the time they are not associated with symptoms, which could be consistent with some women being able to better tolerate these CSTs than others.

To our knowledge, at least four Genome Wide Association Study (GWAS) studies have been conducted to identify the genetic bases for the observed variations in vaginal microbiota composition. All are recent and tend to have limitations. One of the first studies dates from 2020 and involved 359 pregnant women in China to perform a GWAS where the response trait was the presence or absence of specific bacterial species based on 16S RNA data. ^13^ Despite its statistical power and use of microbial DNA data, a limitation of this study is that pregnancy is known to affect vaginal microbiota composition with an increased presence of lactobacilli. ^29^ Another exception comes from a 2021 study in Kenya that performed a GWAS on 171 women, ^12^ using as a response trait the presence or absence of one of three bacterial species, the Shannon diversity, or the CST. In 2024, an analysis was performed in the USA on 686 women living with and at risk for HIV infection, the trait of interest being the presence or absence of bacterial vaginosis (BV). ^11^ However, this analysis was focused on 627 SNPs across 41 genes important in mucosal defense identified from a previous GWAS. Finally, again in 2024, a study performed a GWAS on 12,815 women living in Estonia and identified SNPs associated with recurrent vaginitis. ^14^ Overall, besides their rarity, these earlier studies illustrate the challenge represented by the analysis of microbiota data in human genomics association studies. Most of these rely on symptoms and few consider the genetic diversity of the bacterial population; even though the low species richness of the human vaginal microbiota makes it an ideal system for designing relevant summary statistics.

In this study, we perform a GWAS on 168 women from the PAPCLEAR cohort, which was implemented to analyse human papillomavirus infections, ^17^ using longitudinal vaginal microbiota metabarcoding data (16S) as a response trait. This work is of limited statistical power but stands out in terms of the response traits considered and of the study population.

## Methods

### Data generation

189 participants were enrolled in the PAPCLEAR study, which aimed to understand the human papillomavirus kinetics in the vaginal environment. ^18^

DNA was extracted using the QIAamp ^®^ DNA mini kit (QIAGEN Inc.) from PBMCs extracted from 10 mL of circulating blood following standard protocol for body fluids. Eleven samples were lost during the extraction process, and DNA could not be retrieved.

We then genotyped 168 participants using an Illumina Global Screening Assay Multi-Disease (GSA-MD) genotyping assay, which targets approximately 800,000 SNPs in the genome.

For each of the participants, we used vaginal microbiota 16S metabarcoding data described in an earlier study. ^5^

### Data curation

In order to perform a GWAS, we had to transform the vaginal microbiota data into a single summary statistic. Given the uneven number of samples for each participant and given that prior studies show that a single sample predicts with good accuracy the vaginal microbiota trajectory over the following 18 months, ^6^ we averaged all the visits to obtain a mean dataset for the relative abundance of 372 bacterial species identified from the 16S sequence data using the SpeciateIt software package. ^30^ We analysed this dataset for community state type (CST) composition using the VALENCIA nearest centroid classifier. ^19^ Intuitively, this algorithm uses a large reference dataset (3,160 taxonomic profiles from 1975 women) to compute a distance to reference CSTs for any sample based on the relative frequency of up to 199 bacterial taxa. From this, we could obtain the distance to each centroid, as well as the CST assignment. We used this data to compute two traits: a binary CST assignment (CST-4 *vs.* non-CST-4) and a quantitative shortest distance to CST-4.

Using the R diversity function from the vegan package with the mean relative abundance data, we computed we computed Shannon and Simpson diversity indexes, which are given by the following formula, with *p _i_* denoting the relative frequency of species *i*:

$$D_{\mathrm{sh}}=-\sum_{i\in s}p_{i}\;\ln\left(p_{i}\right)$$

$$D_{\mathrm{si}}=\frac{1}{\sum_{i\in S}p_{i}^{2}}$$

While Shannon’s entropy ( *D _sh_*) is widely used in microbiome research, Simpson’s diversity ( *D _si_*) places more weight on evenness and is, therefore, more sensitive to changes in the low-richness communities.

### Genome Wide Association Study

We performed the association study itself using plink2, using the linear regression with the --covar option to use the first ten MDS components as covariates. Only the results assuming an additive genetic effect were kept, and SNPs with p-values higher than 0.01 were ignored for further analyses.

### SNP imputation

In order to enrich our dataset, we imputed genomic positions not directly covered by the GSA-MD assay using IMPUTE2. ^20^ For this, we downloaded the reference genome v.38 and exported each chromosome from the main file using plink v.2.0. ^21^

### Quality control

We performed classical steps for quality control, as summarised in Ref 22, namely, we:

• removed variants that were too rare or participants with too many missing variants with a 2% threshold;
• removed variants with a minority allele frequency with a 5% threshold;
• checked for a Hardy-Weinberg equilibrium of the variants;
• removed individuals with more than 2 standard deviations in terms of heterozygosity;
• checked for cryptic relatedness, assuming a π threshold value of 0.2.

Using the 1000 Genomes database reference, we also investigated the shared ancestry between our participants. For this, we performed a multidimensional scaling (MDS) clustering, pooling our PAPCLEAR dataset with that from the 1000 Genomes.

The output of this quality control is available in the supplementary HTML file at https://doi.org/10.57745/LGZANK

### Heritability estimation

To help identify the most suitable phenotypic trait for GWAS investigation, we estimated the variance explained by all the SNPs using GCTA, ^23^ which implements a restricted maximum likelihood (REML) approach.

## Results

### Cohort description

The PAPCLEAR study enrolled women aged from 18 to 25 years old, living in the area of Montpellier (France), and who were primarily university students (119/138, 86%) at the time of the study. The main characteristics of the study population have been reported in earlier studies and some are shown in Table 1 for the samples analysed here, with a stratification in terms of vaginal microbiota composition. Most of the associations observed are consistent with earlier studies since women with a CST-4, i.e. *Lactobacillus*-poor vaginal microbiota, report lower condom usage from the male partner, higher lifetime number of sexual partners, and identify themselves less as ‘caucasian’. ^24^ Their body mass index (BMI) and age are also higher, and they have more often been pregnant. In terms of CST differences, the most frequent is CST-1, followed by CST-3, and then CST-4 (which is *Lactobacillus*-poor). As expected, Shannon and Simpson diversity indexes are much lower in the *Lactobacillus*-dominated microbiotas. ^4^

**Table 1.** Cohort profile stratified by vaginal microbiota composition. Numerical variables are centred and scaled. The p-value indicated the outcome of a Fisher exact test for categorical variables and a Kruskal-Wallis rank sum test for continuous variables (the interquartile range is shown in brackets). Significant differences are in italic.

	CST-4	*Lactobacillus*	p-value
Number of participants	19	149	
female affinity (%)	4 (21.1)	26 (17.6)	0.751
male affinity (%)	18 (94.7)	145 (99.3)	0.218
smoker (%)	10 (76.9)	61 (70.9)	0.754
regular sport practice (%)	7 (36.8)	77 (51.7)	0.330
chlamydia infection (%)	0 (0.0)	6 (5.2)	1.000
menstrual cup user (%)	3 (15.8)	24 (16.1)	1.000
vaginal products user (%)	8 (42.1)	69 (46.3)	0.810
tampon user (%)	8 (42.1)	45 (30.2)	0.304
previous pregnancy (%)	3 (15.8)	5 (3.4)	0.049
vaginal douching (%)	1 (5.3)	3 (2.0)	0.384
male partner using condom (%)	4 (21.1)	84 (56.4)	0.006
identifies as Caucasian (%)	11 (57.9)	125 (83.9)	0.012
alcohol consumption	−0.07 [−0.52, 0.39]	−0.07 [−0.52, 0.84]	0.583
body mass index	0.11 [−0.28, 1.23]	−0.35 [−0.73, 0.38]	0.030
age at inclusion	0.23 [0.23, 0.72]	−0.27 [−0.77, 0.72]	0.123
age at first menstruation	−0.18 [−1.11, 0.19]	0.19 [−0.55, 0.93]	0.244
lifetime number of partners	0.30 [−0.38, 1.45]	−0.38 [−0.67, 0.30]	0.020
microbiota Shannon diversity	1.67 [1.01, 2.11]	0.46 [0.22, 0.76]	<0.001
microbiota Simpson diversity	3.46 [2.02, 5.14]	1.30 [1.08, 1.63]	<0.001
CST (%)			<0.001
I.A	0 (0.0)	60 (40.3)	
I.B	0 (0.0)	24 (16.1)	
II	0 (0.0)	2 (1.3)	
III.A	0 (0.0)	39 (26.2)	
III.B	0 (0.0)	19 (12.8)	
IV.A	3 (15.8)	0 (0.0)	
IV.B	15 (78.9)	0 (0.0)	
IV.C	1 (5.3)	0 (0.0)	
V	0 (0.0)	5 (3.4)	

**Figure 1.**
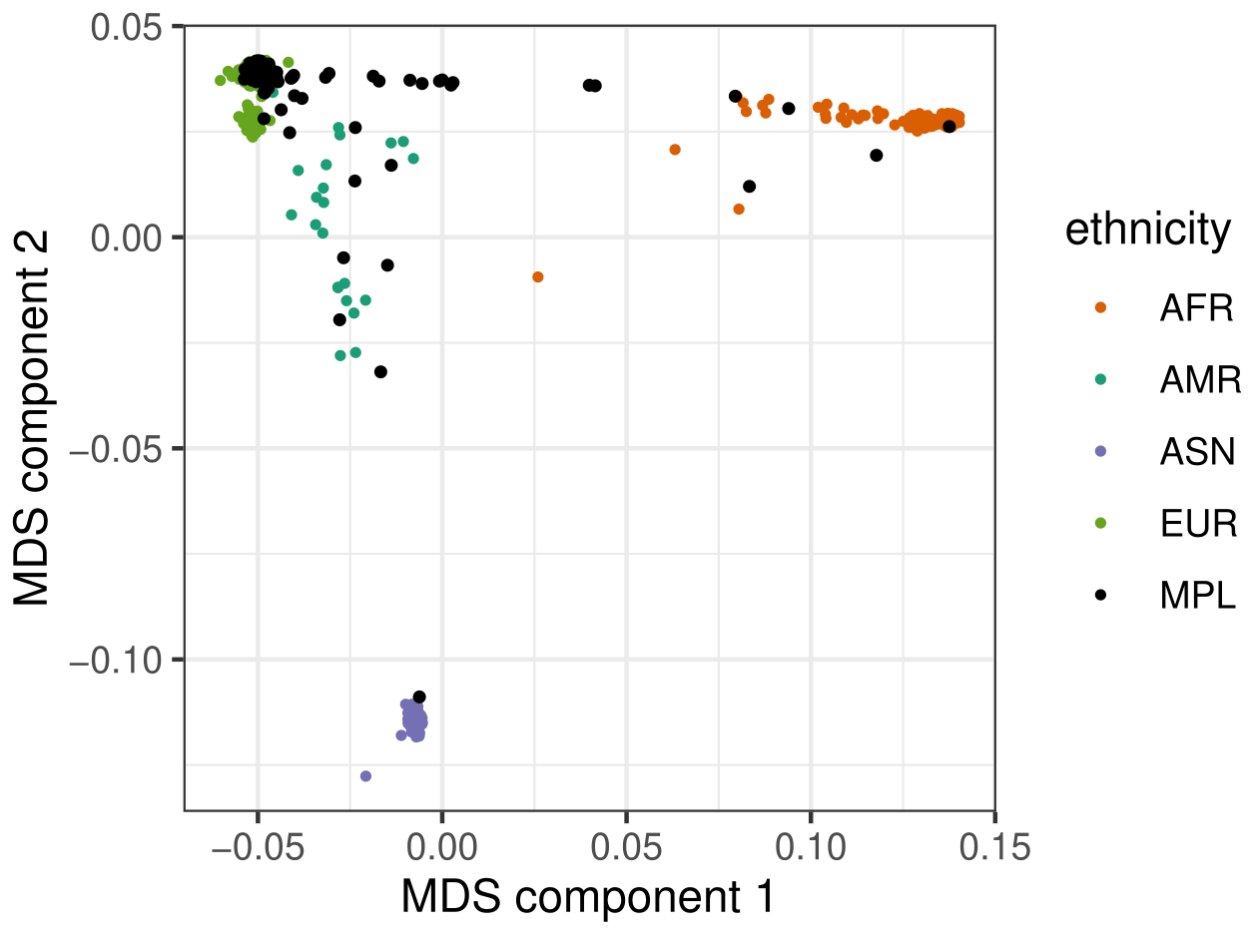
Multidimensional scaling (MDS) plot of population stratification. The four colours show the main ancestries for the 1000 Genomes database. The PAPCLEAR study participants from Montpellier (‘MPL’) are shown in black.

The genetic composition of the large majority of the study population corresponds to the individuals from European ancestry in the 1000 genomes database ( Figure 1). Given the limited size of the sample, we chose not to remove the participants far away from the European-ancestry cluster but used the MDS coordinates as covariates in the association study as a control.

### Genetic control over the traits

**Figure 2.**
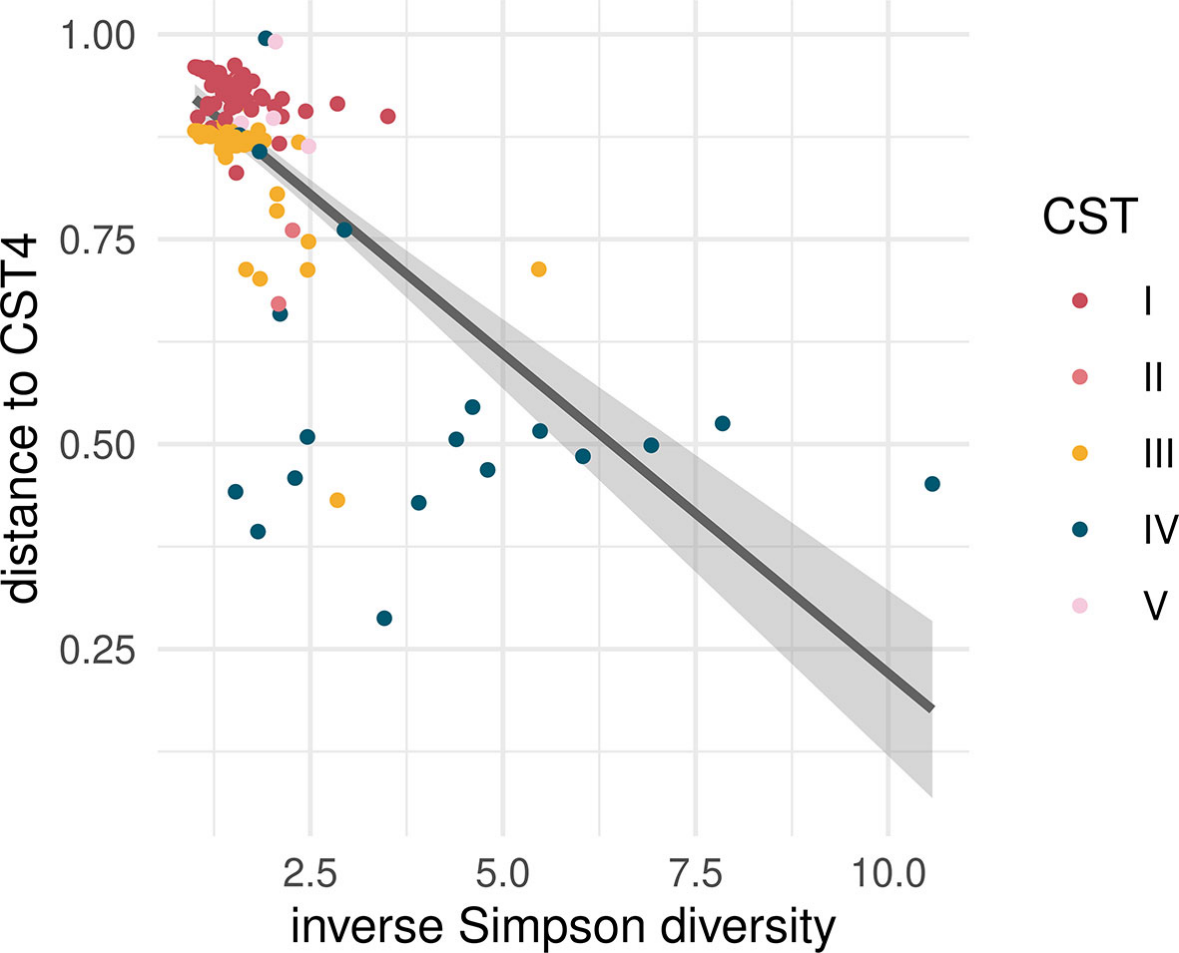
Distribution of the phenotypic traits of interest.

When considering the explanatory power of all the SNPs on of a trait of interest, the signal was limited when using the binary trait (CST-4 *vs.* non-CST-4) with an estimated genetic variance explained by all the SNPs of the genome of 0.09 ( Table 2). For the Shannon diversity, which is highly correlated with the distance to CST-4 ( Figure 2), this genetic variance was 0.24. For the Simpson diversity, it was even higher with a value of 1.80. Therefore, we focus on the latter trait in the following. Note that GCTA found little environmental variance, which is likely due to the small size of our dataset.

**Table 2.** Variance explained by the whole genome for microbiota-related traits. V(G) stands for genetic variance, V(e) for environmental variance, h2 is the fraction of the total variance explained by the genetic variance, log(L) is the log likelihood and P-value the p-value associated with the final estimate for the restricted maximum likelihood (REML) model (see Ref. 23 for details).

Source	CST-4 vs. non-CST-4	Simpson diversity	Shannon diversity
V(G)	0.09	1.80	0.29
V(e)	0.0	0.000003	0.0
h2	0.99	0.99	0.99
log(L)	109.50	−63.95	3.60
p-value	0.0013	0.0008	0.015

Each of the dots corresponds to one of the participants. The line shows the output of the linear model, showing the negative correlation between Simpson diversity and distance to CST-4. Colours show the different CSTs (CST-4 is poor in lactobacilli).

### Association study

**Figure 3.**
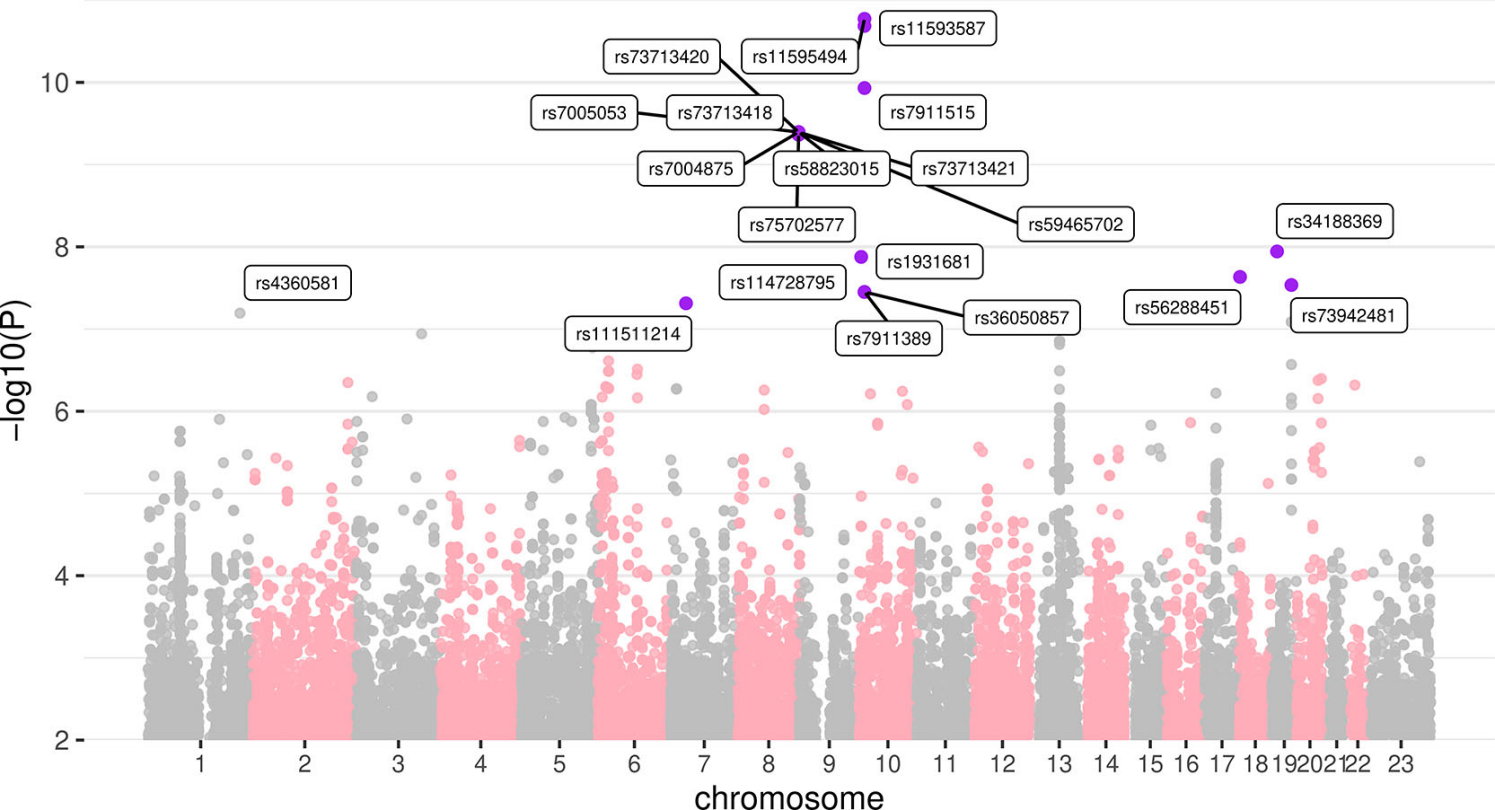
Manhattan plot showing associations with vaginal microbiota Shannon diversity. Ancestry is taken into account in the regression via the MDS components. Colours indicate SNPs or regions of interest, which are also labelled. The X chromosome is labelled as ‘23’.

The genome wide association study, assuming an additive model and using the ten major MDS components as covariates, identified several specific genomic regions as being significantly associated with vaginal microbiota Simpson diversity, assuming a classical 5 × 10–8 threshold for p-value significance ( Figure 3).

The most significant association was with several SNPs located in chromosome 10 and in the gene coding the Coiled-coil domain containing 3 (CCDC3) protein ( Table 3). This secretory is expressed in vascular endothelial cells and has no reported associations with microbiota. The next two most strongly associated regions were located in chromosomes 8 and 19, and far from any human gene. A fourth region of interest was located in a non-coding area of chromosome 10, close to the region coding for the Aldo-keto reductase family 1 member C4 (AKR1C4) protein. The final three significant SNPs are located in chromosomes 18, 19, and 7, and in the coding regions of Elastin microfibril interfacer 2 (EMILIN2), Paraneoplastic antigen-like protein 8B (PNMA8B), and Endonuclease/exonuclease/phosphatase family domain containing 1 (EEPD1) genes. EMILIN2 is an extracellular matrix constituent involved in angiogenesis but with no reported link with microbiota. Finally, another SNP was nearly significant and located in a non-coding region of chromosome 1, close to the gene coding for the Estrogen-related receptor gamma (ESRRG) protein.

**Table 3.** Characteristics of the SNPs significantly associated with vaginal microbiota Simpson diversity. ID is the reference SNP cluster ID, CHR is the chromosome number, BP is the position in the chromosome using genome reference GRCh37, REF is the reference allele, MUT is the alternative allele, MAF is the minority allele frequency in the dataset, EUR freq. and AFR freq. Are the MAF in European and African populations based on the Allele Frequency Aggregator (ALFA) project, Obs. is the number of observations of the position in our dataset, Model is the analysis used (CST is when using CST-4 vs. non-CST-4 as a response trait and Simpson2 is when removing one key participant from the dataset), Beta is the regression coefficient of the model, SE its standard error, and p-val. Its asymptotic p-value. The non-significant value, i.e. with a p-value above 5·10–8 is in italic. ‘coding’ indicates any gene the SNP may be located in and ‘nearby’ indicates any potential gene of interest within less than 6,000 base.

ID	CHR	BP	REF	MUT	MAF	EUR freq.	AFR freq.	Obs.	Model	Beta	SE	p-val.	gene	nearby
rs11595494	10	12,992,365	G	C	0.052	0.050	0.233	163	Simpson	1.80	0.25	1.69e-11	CCDC3	---
rs73713420	8	142,685,979	A	G	0.052	0.020	0.305	164	Simpson	1.91	0.29	4.03e-10	---	---
rs34188369	19	13,293,164	G	A	0.058	0.036	0.253	164	Simpson	1.64	0.27	1.14e-08	---	---
rs1931681	10	5,263,915	G	T	0.053	0.98	0.78	162	Simpson	1.54	0.26	1.33e-08	---	AKR1C4
rs56288451	18	2,909,700	C	T	0.055	0.047	0.133	164	Simpson	1.54	0.26	2.32e-08	EMILIN2	---
rs73942481	19	46,993,058	A	G	0.064	0.054	0.105	164	Simpson	1.43	0.24	2.92e-08	PNMA8B	---
rs111511214	7	36,267,254	G	A	0.053	0.034	0.316	161	Simpson	1.50	0.26	4.87e-08	EEPD1	---
rs4360581	1	216,671,992	C	T	0.049	0.048	0.072	164	Simpson	1.42	0.25	*6.41e-08*	---	ESRRG
rs8081213	17	25,297,867	A	C	0.056	0.069	0.182	161	CST	0.70	0.11	1.32e-08	---	---
rs113602804	10	5,265,452	T	C	0.052	0.004	0.005	163	Simpson2	1.33	0.22	1.70e-08	---	AKR1C4
rs8081213	17	25297867	A	C	0.056	0.069	0.182	161	Shannon	0.70	0.12	2.20E-08	---	---

**Figure 4.**
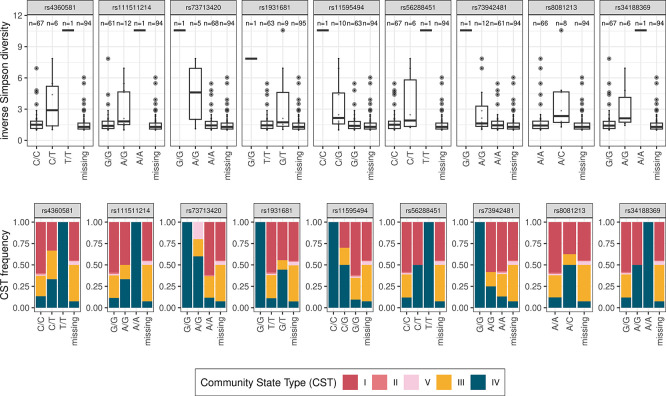
Vaginal microbiota Simpson diversity (top) and community state type (bottom) stratified by genetic variant. On the top panels, boxes show the medians, quartiles, and quantiles. Dots show the outliers. For some individuals, the coverage for the genomic position is missing. Numbers above the boxes indicate group sample sizes.

A closer look at the distribution of these SNPs in the dataset revealed a potential confounding effect from one participant. Indeed, as shown in the top panels of Figure 4, except for rs1931681, this person is homozygote for the minority allele and her microbiota has one of the highest diversity values. Furthermore, as illustrated by a linear model stratified by variant for rs11595494 ( Table 4), the participant reported using vaginal douching, a practice known to be strongly associated with CST-4, i.e. *Lactobacillus*-poor vaginal microbiota, but rare in our cohort ( Table 1). Incidentally, note that we did not detect any other significant difference in this analysis.

**Table 4.** Cohort profile stratified by mutation at rs11595494. See Table 1 for details.

	C/C	C/T	T/T	p
number of participants	67	6	1	
female affinity (%)	12 (17.9)	1 (16.7)	0 (0.0)	1.000
male affinity (%)	67 (100.0)	6 (100.0)	1 (100.0)	NA
smoker (%)	26 (63.4)	6 (100.0)	0 (NaN)	0.157
regular sport practice (%)	36 (53.7)	2 (33.3)	0 (0.0)	0.305
menstrual cup user (%)	15 (22.4)	0 (0.0)	0 (0.0)	0.469
vaginal products user (%)	30 (44.8)	3 (50.0)	0 (0.0)	1.000
tampon user (%)	18 (26.9)	1 (16.7)	0 (0.0)	1.000
previous pregnancy (%)	3 (4.5)	1 (16.7)	0 (0.0)	0.334
*vaginal douching (%)*	*1 (1.5)*	*0 (0.0)*	*1 (100.0)*	*0.033*
male partner using condom (%)	34 (50.7)	3 (50.0)	0 (0.0)	1.000
alcohol consumption	−0.07 [−0.52, 0.61]	−0.52 [−0.52, −0.18]	−1.43 [−1.43, −1.43]	0.235
BMI	−0.25 [−0.57, 0.67]	0.33 [−0.43, 1.15]	−0.64 [−0.64, −0.64]	0.385
age at inclusion	0.72 [−0.27, 0.72]	0.72 [0.35, 0.72]	−1.76 [−1.76, −1.76]	0.209
age at first mentruations	0.19 [−0.55, 0.93]	−0.18 [−1.11, 0.19]	0.19 [0.19, 0.19]	0.482
number of sexual partners	−0.19 [−0.62, 0.30]	0.73 [−0.19, 1.79]	0.49 [0.49, 0.49]	0.238

Our analysis controls for shared ancestry by using the principal components of the MDS clustering but we performed additional analyses to investigate the robustness of our results. First, we redid the previous analysis without the aforementioned participant. Only one of the regions remained significant with an SNP located in chromosome 10 close to the AKR1C4 gene (model “Simpson2” in Table 2). Furthermore, we performed a multivariate linear regression using the Simpson diversity as a response variable, and, as explanatory variables, the main variants at seven genomic positions, the covariates associated with vaginal microbiota composition (in Table 1), and the first ten components of the MDS. As shown in Table 5, the only variables significantly associated with Simpson diversity were the SNPs and the addition of the behavioural covariates only marginally improved the percentage of the variance explained (the adjusted R-squared increased only from 0.63 to 0.65).

**Table 5.** Linear model with socio-demographic covariates. ‘ref.’ indicates the reference value for categorical variables, beta is the effect term, and Ci stands for confidence interval. MDS PC indicates the principal components of the multidimensional scaling clustering (see the main text). The adjusted R-squared of the model is 0.65 (and 0.63 without the behavioural covariates).

	Beta	95% CI	p-value
rs4360581 (ref. C/C)			
*C/T*	1.2	0.39, 2.0	0.004
*missing*	−0.10	−1.8, 1.6	>0.9
*T/T*	7.6	5.3, 9.8	<0.001
Rs111511214 (ref. A/A)			
*A/G*	0.53	−0.09, 1.1	0.095
rs73713420 (ref. A/A)			
*A/G*	1.1	0.14, 2.0	0.025
rs1931681 (ref. G/G)			
*G/T*	−3.0	−5.1, −0.98	0.004
*T/T*	−2.9	−5.0, −0.84	0.006
*missing*	−2.6	−5.3, 0.07	0.056
rs11595494 (ref. C/C)			
*C/G*	0.66	0.05, 1.3	0.034
rs56288451 (ref. C/C)			
*C/T*	0.70	−0.06, 1.5	0.072
rs73942481 (ref. A/A)			
*A/G*	0.12	−0.52, 0.76	0.7
rs8081213 (ref. A/A)			
*A/C*	0.60	−0.16, 1.4	0.12
rs34188369 (ref. A/A)			
*A/G*	1.2	0.42, 2.0	0.003
MDS PC1	2.3	−0.79, 5.5	0.14
MDS PC2	0.59	−5.4, 6.6	0.8
MDS PC3	9.8	−1.8, 21	0.10
MDS PC4	−2.6	−10, 5.3	0.5
MDS PC5	−6.4	−43, 30	0.7
MDS PC6	−13	−32, 6.6	0.2
MDS PC7	8.1	−7.1, 23	0.3
MDS PC8	−16	−42, 9.4	0.2
MDS PC9	−32	−67, 3.0	0.072
MDS PC10	−6.2	−41, 28	0.7
body mass index	0.12	−0.01, 0.25	0.078
regular sport practice (ref. no)	−0.14	−0.40, 0.12	0.3
age at first menstruation	0.05	−0.08, 0.18	0.5
condom use (ref. no)	−0.12	−0.37, 0.13	0.3
previous pregnancy (ref. no)	−0.32	−0.99, 0.35	0.3
age at inclusion	−0.01	−0.15, 0.13	>0.9

For completeness, we also performed GWAS using the Shannon diversity index or the CST composition (CST-4 vs. non-CST-4). In both models, a non-coding region from chromosome 17 was found to be significantly associated with the trait.

## Discussion

Few studies have explored associations between human genetics and the vaginal microbiota. Most of these rely on symptoms rather than genetic microbiological data. Furthermore, few studies have been conducted on women of European ancestry.

Building on data collected during the PAPCLEAR study, ^5^ we performed a GWAS using the Simpson diversity index of the vaginal microbiota as our trait of interest. Our microarray allowed us to identify 320,452 variants, which we enriched to 2,927,125 variants using imputation methods, and the analysis revealed several SNPs of interest, most of which were in or close from coding regions of the human genome. However, the mechanistic links between the genes involved and the vaginal microbiome are limited except for the two cases where the SNPs were close from the gene coding region. The first one, AKR1C4, has been shown to be associated with inflammatory bowel disease. ^31^ This protein is also expected to strongly interact with proteins involved in the degradation of testosterone and progesterone (SDR5A1 and SDR5A2) according to the STRING prediction algorithm. ^32^ The second one, ESRRG is involved in estrogen signaling and interacts with other hormone receptor proteins. It has also been shown to be involved in bacterial infections by *Helicobacter pylori.* ^33^

When using Shannon diversity or a binary trait (CST-4 vs. non-CST-4) as the response variable in our model, we identified another genomic area that was not associated with Simpson diversity. We do expect Shannon diversity and the CST stratification to be more alike than the Simpson diversity, and the SNPs identified with the former were in non-coding regions, which makes it difficult to provide more mechanistic interpretations.

An additional issue is that most of the SNPs significantly associated with Simpson diversity were conditioned by the inclusion in the dataset of a specific participant. We decided to report these results because our analysis takes into account genetic ancestry and because reporting these SNPs makes it possible for future, more powered, studies to confirm or refute these associations. Note also that the SNPs close from the AKR1C4 gene remained significantly associated with Simpson diversity when the participant was removed from the dataset.

In addition to the number of participants, our study is also limited in terms of genome coverage. The variants we find do not seem to be correlated with covariates classically involved in vaginal microbiota differences and a deeper sequencing of the human genome could identify positions that were not sufficiently covered in the assay used.

Performing a GWAS on microbiota raises several challenges by definition. A first issue has to do with the temporal variability. In the case of the vaginal microbiota, earlier studies show that the communities are highly stable on a weekly basis ^5^ and that a single time point single sample predicts with good accuracy the vaginal microbiota trajectory over the following 18 months. ^6^ To further buffer potential variations, we averaged all the observations of a single participant to define the observed trait. Another possibility could have been to analyse the temporal variability of the vaginal microbiota. However, this would have been problematic because the number of observations per participant was highly variable, some being followed for less than two months and others for more than two years. ^5^ A second issue has to do with the trait used to describe the microbiota. The high level of clustering of vaginal microbiota samples offers several options to address this problem. One of them is to focus on the most abundant species, e.g. using the Shannon diversity index or the presence/absence of lactobacilli, whereas the other is to put more importance on evenness in species abundance, e.g. using the Simpson diversity index. We show that the latter yields much more variance across samples and is associated with more genomic regions. With more homogeneous follow-ups between participants and a larger cohort size, a possibility could have been to perform a hierarchical clustering on the follow-ups themselves to include a third category of women who alternate between CSTs. ^5^^,^^6^ Nevertheless, compared to other microbiota, the temporal stability and strong structure of the vaginal microbiota make it an ideal study system to investigate the coevolution between hosts and their microbiota.

## Ethics

The PAPCLEAR study has been approved by the Comitee of Persons Protection (Comité de Protection des Personnes, CPP) Sud Méditerranée I on 25 April 2016 (reference number 2016-A00712–49); by the Consultative Comitee on Information processing in the context of research in health (Comité Consultatif sur le Traitement de l’Information en matière de Recherche dans le domaine de la Santé, reference number 16.504); by the National Commission on Informatics and Freedom (Commission Nationale Informatique et Libertés, reference number MMS/ABD/AR1612278, decision number DR-2016-488), by the National Agency of Security of Drugs and Health Products (Agence Nationale de Sécurité du Médicament et des Produits de Santé, reference 20160072000007), and is registered at ClinicalTrials.gov under the ID NCT02946346. All participants provided written informed consent to participate in the study.

## Data Availability

The output of the GWAS for Shannon diversity, the metadata, and the output of the quality control analysis can be accessed on the *Recherche Data Gouv* data repository at https://doi.org/10.57745/LGZANK, along with the scripts used to generate the figures and table in the manuscript. ^26^ It is also available on the Open Science Framework DOI 10.17605/OSF.IO/MZTWX. ^27^ Creative Commons Attribution 4.0 International Public License.
