# Supplementary material for: Genome wide association study of vaginal microbiota genetic diversity in French women
Source: Open Res Eur. 2026 Mar 11;5:186. Originally published 2025 Jul 15. [Version 2] doi: 10.12688/openreseurope.20462.2 (PMC13003221; doi:10.12688/openreseurope.20462.2)
Supplement: Peer review history

# Reviewer report: Reviewer response for version 2

**DOI:** 10.21956/openreseurope.24881.r71043

**Published:** 2026-03-19

**Authors:** Luisa W. Hugerth

**Affiliations:** 1 Uppsala University, Uppsala, Sweden

The authors have thoroughly addressed all my previous concerns.

Is the study design appropriate and does the work have academic merit?

Partly

Is the work clearly and accurately presented and does it cite the current literature?

Yes

If applicable, is the statistical analysis and its interpretation appropriate?

Yes

Are all the source data underlying the results available to ensure full reproducibility?

Partly

Are the conclusions drawn adequately supported by the results?

Partly

Are sufficient details of methods and analysis provided to allow replication by others?

Partly

Reviewer Expertise:

Microbiome; Women's health

I confirm that I have read this submission and believe that I have an appropriate level of expertise to confirm that it is of an acceptable scientific standard.

# Reviewer report: Reviewer response for version 2

**DOI:** 10.21956/openreseurope.24881.r71042

**Published:** 2026-03-19

**Authors:** Antonio Charlys da Costa, Tania Regina Tozetto-Mendoza

**Affiliations:** 1 University of São Paulo, São Paulo, Brazil; 2 Virology Lab, Universidade de Sao Paulo Instituto de Medicina Tropical de Sao Paulo, São Paulo, State of São Paulo, Brazil

I consider the article ready for indexing

Is the study design appropriate and does the work have academic merit?

Yes

Is the work clearly and accurately presented and does it cite the current literature?

Partly

If applicable, is the statistical analysis and its interpretation appropriate?

Partly

Are all the source data underlying the results available to ensure full reproducibility?

Yes

Are the conclusions drawn adequately supported by the results?

Yes

Are sufficient details of methods and analysis provided to allow replication by others?

Partly

Reviewer Expertise:

Metagenomics and virus discovery

We confirm that we have read this submission and believe that we have an appropriate level of expertise to confirm that it is of an acceptable scientific standard.

# Reviewer report: Reviewer response for version 1

**DOI:** 10.21956/openreseurope.22142.r57586

**Published:** 2025-09-01

**Authors:** Luisa W. Hugerth

**Affiliations:** 1 Uppsala University, Uppsala, Sweden

Alizon and colleagues set out to identify human genetic loci associated with the vaginal microbiome. This is interesting for two reasons. Firstly, the vaginal microbiome has been previously associated with many adverse obstetric and gynecologic outcomes. While in some cases there are clear mechanistic evidence for it, it is equally possible that the microbiome is (partly) a biomarker for an internal underlying process, e.g. connected to the immune system or to mucosal integrity. Secondly, abundant data from around the globe shows a stratification of the vaginal microbiome in regards to ethnicity, as well as diverging risk patterns for some microbes in different populations. For all these reasons, the present research has microbial ecological and medical interest.

 However, defining a "microbiome" as a phenotype is a complicated endeavour, and could be improved in the current work. Additionally, the treatment of confounders is not entirely clear, as detailed below:

 1. Data availability and methods. 

 https://entrepot.recherche.data.gouv.fr/dataset.xhtml?persistentId=doi:10.57745/LGZANK contains important summary data, but I wouldn't call it raw data. At least for the microbiome, raw fastq reads (depleted of human DNA) should be submitted to ENA, together with appropriate patient-level data. 

 Related to this, even if the 16S data is previously published, please provide minimal information on it in the methods, namely: hypervariable region amplified, platform used for clustering or denoising (Deblur, DADA2, Unoise, other?) and database+algorithm used for taxonomic assignment.

 Additionally, the provided link http://www.doi.org/10.17605/OSF.IO/MZTWX redirects me to the baseline page https://osf.io/, so I'm not sure what should be in it.

 2. Demographics and other confounders.

 Since the data in table 1 is centralized and standardized, I have no clue whether the participants included are school-age, reproductive age or postmenopausal. This should be stated more clearly.

 Additionally, it would be important to provide the median and range for the number of samples collected for each participant, since this may impact the averaging procedure. Related to this, there is strong shaping of the microbiome in relation to the menstrual cycle (see e.g. https://pubmed.ncbi.nlm.nih.gov/39160615/). Is there any information on the participants cycle (assuming they are all pre-menopausal?) Is averaging all samples the best approach? Or would it make more sense to look for "mid-cycle" samples (and then average these)? At any rate, more information on the menstrual cycle would be needed for data interpretation.

 Furthermore, parity is another very strong predictor of the vaginal microbiome, and there is a significant imbalance in gravidity at least for one locus. Excluding the 8 women that have previously been pregnant (as well as any partipants aged 45+) may give a cleaner signal.

 To be clear, I would love to see a similar study as yours focusing on middle-aged women, but from what I can tell from your data, you're not powered for it; and therefore I would advise on focusing on the nulliparous 18-35-years-old and really getting that signal out.

 3. Defining dysbiosis.

 This is of course a very complicated endeavour, and I appreciate that you have attempted a variety of methods, focusing the GWAS on the most promising. Still, there are some things that could be improved in this area, as follows:

 VALENCIA centroids are based on a number of pre-defined taxa. How well did your taxa match VALENCIA's? Were species not matching VALENCIA excluded (and the data re-normalized) or were they kept? Describe the procedure a bit more clearly in the methods.

 VALENCIA also has a coding quirk that, if no CST are of high confidence, defaults to CST-I. Were there any cutoffs for CST assignment? How did you handle low-confidence assignments? Please specify in the methods. Would it make sense to have them as putative CST-IV, in the sense of "not dominated by any of the 4 cannonical Lacto species"?

 Conversely, were there any samples dominated by a non-canonical Lacto, such as L. vaginalis or Limosilactobacillus spp? How were these handled? VALENCIA would likely not be able to assign them with any confidence, but they strike me as definitely not CST-IV.

 CST-IV is, as you know, highly polymorphic. Indeed, you seem to have 15 individuals with CST IV-B, 3 CST IV-A and 1 CST IV-C. Is it possible that you would get a cleaner GWAS signal by focusing on CST IV-B, and perhaps excluding the other 4 individuals as neither case nor control?

 Relatedly, how come a participant with CST-IVC is placed in table 1 as "Lactobacillus-dominated"? How was Lacto-dominance determined, if not through CST? Please specify in the methods.

 If all of this cleaning doesn't improve the CST-IV signal (or if you decide not to pursue it), it may still be productive to improve the diversity signal. While Shannon's entropy is widely used in microbiome research, Simpson's diversity (which places more weight on evenness) is, in my experience, more sensitive to changes in the low-richness vaginal microbiome. Alternatively, you may find it productive to break down the global diversity measure into its components of richness and evenness. There could be quite different processes controlling richness (the establishment of new species, even at a very small number) and evenness (the possibility for one species to thoroughly dominate the vaginal microbiome).

 All in all, this is a very interesting paper. Small adjustments described above could really make the conclusions much more convincing and harder to dismiss by competing teams.

Is the study design appropriate and does the work have academic merit?

Partly

Is the work clearly and accurately presented and does it cite the current literature?

Yes

If applicable, is the statistical analysis and its interpretation appropriate?

Yes

Are all the source data underlying the results available to ensure full reproducibility?

Partly

Are the conclusions drawn adequately supported by the results?

Partly

Are sufficient details of methods and analysis provided to allow replication by others?

Partly

Reviewer Expertise:

Microbiome; Women's health

I confirm that I have read this submission and believe that I have an appropriate level of expertise to confirm that it is of an acceptable scientific standard, however I have significant reservations, as outlined above.

## References

[1] : Defining Vaginal Community Dynamics: daily microbiome transitions, the role of menstruation, bacteriophages, and bacterial genes. *Microbiome* .2024;12(1) : 10.1186/s40168-024-01870-5 10.1186/s40168-024-01870-5 39160615 PMC11331738

# Response

**Published:** 2026-01-27

**Authors:** Samuel Alizon

**Affiliations:** Center for Interdisciplinary Research in Biology, Centre National de la Recherche Scientifique, Paris, Île-de-France, France

*Alizon and colleagues set out to identify human genetic loci associated with the vaginal microbiome. This is interesting for two reasons. Firstly, the vaginal microbiome has been previously associated with many adverse obstetric and gynecologic outcomes. While in some cases there are clear mechanistic evidence for it, it is equally possible that the microbiome is (partly) a biomarker for an internal underlying process, e.g. connected to the immune system or to mucosal integrity. Secondly, abundant data from around the globe shows a stratification of the vaginal microbiome in regards to ethnicity, as well as diverging risk patterns for some microbes in different populations. For all these reasons, the present research has microbial ecological and medical interest.*

*However, defining a "microbiome" as a phenotype is a complicated endeavour, and could be improved in the current work. Additionally, the treatment of confounders is not entirely clear, as detailed below:*

 Reply: Thank for the positive appreciation of our work! We completely agree that finding an appropriate summary statistics to characterise the microbiote is challenging. _______________________________________________________________________________________ *Q 2.1 Data availability and methods. https://entrepot.recherche.data.gouv.fr/dataset.xhtml?persistentId=doi:10.57745/LGZANK contains important summary data, but I wouldn’t call it raw data. At least for the microbiome, raw fastq reads (depleted of human DNA) should be submitted to ENA, together with appropriate patient-level data.*

*Related to this, even if the 16S data is previously published, please provide minimal information on it in the methods, namely: hypervariable region amplified, platform used for clustering or denoising (Deblur, DADA2, Unoise, other?) and database+algorithm used for taxonomic assignment.*

*Additionally, the provided link http://www.doi.org/10.17605/OSF.IO/MZTWX redirects me to the baseline page https://osf.io/, so I’m not sure what should be in it.*

 Reply: We are still in the process of recovering the raw data for the upload in ENA. The process is taking some time because half of the files were archived. This also partly explains the delay in resubmitting. Hopefully this can be solved in the next weeks, but we preferred to resubmit to avoid wasting even more time. We added details about the generation of the 16S data in the methods from our previous publication. We also now mention summary data instead of raw data to avoid ambiguities. Regarding the link, this is normal: the Open Science Foundation creates a DOI associated with the dataset (this was asked by Open Research Europe because they do not recognise the data.gouv.fr data warehouse). ________________________________________________________________________________________ *Q 2.2 Demographics and other confounders. Since the data in table 1 is centralized and standardized, I have no clue whether the participants included are school-age, reproductive age or postmenopausal. This should be stated more clearly.*

*Additionally, it would be important to provide the median and range for the number of samples collected for each participant, since this may impact the averaging procedure. Related to this, there is strong shaping of the microbiome in relation to the menstrual cycle (see e.g. https://pubmed.ncbi.nlm.nih.gov/39160615/). Is there any information on the participants cycle (assuming they are all pre-menopausal?) Is averaging all samples the best approach? Or would it make more sense to look for "mid-cycle" samples (and then average these)? At any rate, more information on the menstrual cycle would be needed for data interpretation.*

*Furthermore, parity is another very strong predictor of the vaginal microbiome, and there is a significant imbalance in gravidity at least for one locus. Excluding the 8 women that have previously been pregnant (as well as any partipants aged 45+) may give a cleaner signal.*

*To be clear, I would love to see a similar study as yours focusing on middle-aged women, but from what I can tell from your data, you’re not powered for it; and therefore I would advise on focusing on the nulliparous 18-35-years-old and really getting that signal out.*

 Reply: Thank you for pointing out the fact that the normalisation of the values make them difficult to interpret. We now present more clearly the demographics. About the multiple samples per participants, we refer to the figure from our previous publication. Information about the menstrual cycle was not available and, as we now explain in further details, we pooled the data from each participant. Regarding the 8 participants who reported earlier pregnancies, we indeed find a significant difference in terms of vaginal microbiota composition with slightly less Lactobacillus-rich compositions, although if we correct for multiple hypothesis testing it becomes non significant. We hesitated to perform the GWAS on a restricted dataset but decided not to for two reasons. First, thanks to your suggestion to focus on other diversity metrics, we now have a much stronger signal. Second, there are other factors that may bias our populations of women from 18 to 25, and some have even stronger impact on vaginal microbiota (e.g. the lifetime number of sexual partners). If we attempt to correct for all of these, we will be left with a tiny cohort. Therefore, we decided to maintain our post hoc analysis to check whether these factors differ significantly when stratifying our populations with respect to the allele found at positions of interest. _______________________________________________________________________________________ *Q 2.3 Defining dysbiosis. This is of course a very complicated endeavour, and I appreciate that you have attempted a variety of methods, focusing the GWAS on the most promising. Still, there are some things that could be improved in this area, as follows:*

*VALENCIA centroids are based on a number of pre-defined taxa. How well did your taxa match VALENCIA’s? Were species not matching VALENCIA excluded (and the data re-normalized) or were they kept? Describe the procedure a bit more clearly in the methods.*

*VALENCIA also has a coding quirk that, if no CST are of high confidence, defaults to CST-I. Were there any cutoffs for CST assignment? How did you handle low-confidence assignments? Please specify in the methods. Would it make sense to have them as putative CST-IV, in the sense of "not dominated by any of the 4 cannonical Lacto species"?*

*Conversely, were there any samples dominated by a non-canonical Lacto, such as L. vaginalis or Limosilactobacillus spp? How were these handled? VALENCIA would likely not be able to assign them with any confidence, but they strike me as definitely not CST-IV.*

*CST-IV is, as you know, highly polymorphic. Indeed, you seem to have 15 individuals with CST IV-B, 3 CST IV-A and 1 CST IV-C. Is it possible that you would get a cleaner GWAS signal by focusing on CST IV-B, and perhaps excluding the other 4 individuals as neither case nor control?*

 Reply: We now present the VALENCIA algorithm in further details but it is based on 13,160 taxonomic profiles from 1975 women, meaning that the external database is quite large. Regarding the matching to the database, we can investigate the distance to the CSTs (although these represent "idealised" communities and not actual samples). About the assignment, we are not aware of any ‘default’ assignment to CST-1. For example, one of the samples that seems difficult to attribute to any CST is labelled as a CST5. Other GWAS have considered species diversity in detail (e.g. in China). However, we are unsure that this can be done by correctly controling for multiple hypothesis testing. Furthermore, the size of our cohort strongly limits our possibility to go beyond illustrative results. Finally, we wish to stress that the discussion on the CSTs is somehow secondary since our main results do not involve these. We merely use them to show that an asset of the vaginal microbiota is that there is high correlation between the metric used (although, as you intuited, Simpson diversity yields more signal).  

*Relatedly, how come a participant with CST-IVC is placed in table 1 as "Lactobacillus-dominated"? How was Lacto-dominance determined, if not through CST? Please specify in the methods.*

 Reply: Thank you so much for your careful reading!!! This actually led us to realise that there was a column swap in the data. We reran the whole averaging script and triple checked the outputs. In the end, besides this line, there were only a few very minor differences in diversity metrics that did not change the results.  

*If all of this cleaning doesn’t improve the CST-IV signal (or if you decide not to pursue it), it may still be productive to improve the diversity signal. While Shannon’s entropy is widely used in microbiome research, Simpson’s diversity (which places more weight on evenness) is, in my experience, more sensitive to changes in the low-richness vaginal microbiome. Alternatively, you may find it productive to break down the global diversity measure into its components of richness and evenness. There could be quite different processes controlling richness (the establishment of new species, even at a very small number) and evenness (the possibility for one species to thoroughly dominate the vaginal microbiome).*

 Reply: Again, thank you so much for your helpful comments! We followed your advice and reran all the analyses using Simpson diversity. As you will see in the revised version of the manuscript, we were amazed to see how this lit up the Manhattan plot, which many significant associations. One limitation is that most of the SNPs detected seem to rely on a single participant, whose ancestry differs from the majority of that of the population. When performing the analysis without this participant, only a single SNP remained. We discussed about which results to show in the main text and decided to keep the full dataset for two reasons. First, if we do not report it, future studies will not be able to test these candidate SNPs. Second, we corrected for shared ancestry in the GWAS using 10 components of the MDS clustering so, in statistically, this should be controlled for.

*All in all, this is a very interesting paper. Small adjustments described above could really make the conclusions much more convincing and harder to dismiss by competing teams.*

 Thank you so much for your feedback and the insightful suggestion to look at Simpson diversity!

# Reviewer report: Reviewer response for version 1

**DOI:** 10.21956/openreseurope.22142.r57592

**Published:** 2025-08-13

**Authors:** Antonio Charlys da Costa

**Affiliations:** 1 University of São Paulo, São Paulo, Brazil

This manuscript presents a significant and novel investigation into the genetic basis of vaginal microbiota diversity using GWAS approaches within a European cohort. The utilization of Shannon diversity from 16S metabarcoding data as a quantitative trait constitutes a novel approach that is substantiated by heritability estimates. However, the study's impact is constrained by its relatively modest sample size and the absence of genome-wide significant findings. The functional interpretation of the associated genomic regions remains speculative, and the averaging approach for longitudinal microbiota data may mask critical temporal variation influencing host-microbiota interactions. With moderate revisions aimed at clarifying the methodology, better addressing the limitations, and improving the presentation, this study would contribute to the emerging field of microbiome genetics. Subsequent, more extensive studies with more comprehensive genomic coverage and meticulous control of environmental confounders are necessary to validate and expand upon these findings.

 The abstract could better emphasize the study's limitations, such as sample size and statistical power. It would be beneficial to explicitly state the novelty of using microbial genetic data rather than symptoms earlier in the abstract.

 The extraneous background information from previous GWAS studies could be streamlined to reduce redundancy. In order to avoid the potential for overgeneralization, it would be advisable to enhance the referencing and clarification of certain statements pertaining to ethnicity and behavioral factors. A brief discussion of the challenges and opportunities inherent in the utilization of 16S metabarcoding data for the purpose of defining traits in the context of GWAS is warranted.

 Further elaboration is necessary to clarify the averaging approach for longitudinal microbiota samples. The utilization of the mean as a metric has the potential to obfuscate the temporal dynamics inherent in the data. The rationale for imputation and its effect on downstream analyses could be expanded. There has been a paucity of discourse regarding the implementation of strategies for the control of potential confounders, including but not limited to sexual behavior, antibiotic utilization, and hormonal status.

 The strongest associations do not reach genome-wide significance, a fact that merits greater discussion. The genomic regions identified are distant from known coding or regulatory regions, resulting in a speculative functional interpretation. Due to the modest sample size, the findings must be interpreted with caution and require replication in larger cohorts to ensure validity. The tables that summarize genotype-phenotype associations are not clearly formatted, and the clarity of these tables could be improved.

 A more fruitful discussion would be one that integrates findings with those from previous GWAS or microbiome studies. A more critical examination is necessary to determine the impact of averaging temporal data on genetic associations. The potential influence of environmental or behavioral factors on vaginal microbiota diversity warrants further investigation. The recommendations for future research are somewhat generic; more specific recommendations would improve impact.

 It has been noted that certain sections of the text contain dense jargon, which may be more effectively mitigated through the implementation of simpler language in certain sections. Minor typographical errors and inconsistencies in terminology (e.g., reference to CST4 vs. CST-4) occur on occasion. The inclusion of more informative legends and clearer labeling would enhance the comprehensibility of the figures. It is recommended that the formatting of select tables be revised to enhance clarity, particularly those that present cohort characteristics.

Is the study design appropriate and does the work have academic merit?

Yes

Is the work clearly and accurately presented and does it cite the current literature?

Partly

If applicable, is the statistical analysis and its interpretation appropriate?

Partly

Are all the source data underlying the results available to ensure full reproducibility?

Yes

Are the conclusions drawn adequately supported by the results?

Yes

Are sufficient details of methods and analysis provided to allow replication by others?

Partly

Reviewer Expertise:

Metagenomics and virus discovery

I confirm that I have read this submission and believe that I have an appropriate level of expertise to confirm that it is of an acceptable scientific standard, however I have significant reservations, as outlined above.

# Response

**Published:** 2026-01-27

**Authors:** Samuel Alizon

**Affiliations:** Center for Interdisciplinary Research in Biology, Centre National de la Recherche Scientifique, Paris, Île-de-France, France

*This manuscript presents a significant and novel investigation into the genetic basis of vaginal microbiota diversity using GWAS approaches within a European cohort. The utilization of Shannon diversity from 16S metabarcoding data as a quantitative trait constitutes a novel approach that is substantiated by heritability estimates. However, the study’s impact is constrained by its relatively modest sample size and the absence of genome-wide significant findings. The functional interpretation of the associated genomic regions remains speculative, and the averaging approach for longitudinal microbiota data may mask critical temporal variation influencing host-microbiota interactions. With moderate revisions aimed at clarifying the methodology, better addressing the limitations, and improving the presentation, this study would contribute to the emerging field of microbiome genetics. Subsequent, more extensive studies with more comprehensive genomic coverage and meticulous control of environmental confounders are necessary to validate and expand upon these findings.*

 Reply: Thank you for this accurate summary of our work! _______________________________________________________________________________________ *Q 1.1 The abstract could better emphasize the study’s limitations, such as sample size and statistical power. It would be beneficial to explicitly state the novelty of using microbial genetic data rather than symptoms earlier in the abstract.*

 Reply: We updated the abstract to mention these points. More precisely, we stress that earlier studies tend to use phenotypic traits as proxy instead of microbial DNA in the Background and we mention the size limitation explicitly in our conclusion. ________________________________________________________________________________________ *Q 1.2 The extraneous background information from previous GWAS studies could be streamlined to reduce redundancy. In order to avoid the potential for overgeneralization, it would be advisable to enhance the referencing and clarification of certain statements pertaining to ethnicity and behavioral factors. A brief discussion of the challenges and opportunities inherent in the utilization of 16S metabarcoding data for the purpose of defining traits in the context of GWAS is warranted.*

 Reply: We restructured the introduction to improve the reading. We now first present the general context. Then we grouped all the elements about the vaginal microbiota and its known association with self-reported ethnicity. Next, we discuss in the additional details what, to our knowledge, are the only four studies that have tried to identify human genetic bases for variations in vaginal microbiota composition. Finally, we introduce the present study and stress the challenge associated with using 16S metabarcoding data. ________________________________________________________________________________________ *Q 1.3 Further elaboration is necessary to clarify the averaging approach for longitudinal microbiota samples. The utilization of the mean as a metric has the potential to obfuscate the temporal dynamics inherent in the data. The rationale for imputation and its effect on downstream analyses could be expanded. There has been a paucity of discourse regarding the implementation of strategies for the control of potential confounders, including but not limited to sexual behavior, antibiotic utilization, and hormonal status.*

 Reply: Thank you for pointing this out since summarising microbiota data is indeed a delicate step in a GWAS. Building on the introduction, we now further explain why we decided to average the values from each participant. Ideally, we would have liked to perform a hierarchical clustering on the whole follow-up. Unfortunately, the number of samples per participant was highly variable. However, based on the study from Tamarelle et al. (2024), which we now cite explicitly in the methods, this would likely have led to similar results since they show that a single sample is highly informative for an 18-month trajectory. Regarding the confounders, we now stress that these are important because there are known associations with vaginal microbiota composition. We also explain how we chose the confounders analysed in the study. _______________________________________________________________________________________ *Q 1.4 The strongest associations do not reach genome-wide significance, a fact that merits greater discussion. The genomic regions identified are distant from known coding or regulatory regions, resulting in a speculative functional interpretation. Due to the modest sample size, the findings must be interpreted with caution and require replication in larger cohorts to ensure validity. The tables that summarize genotype-phenotype associations are not clearly formatted, and the clarity of these tables could be improved.*

 Reply: We now explicitly mention when some results are non-significant. We also rephrased the opening of the Discussion to stress all these limitations. However, note that thanks to the insightful suggestion from Reviewer #2 to use Simpson diversity, most of the SNPs we find are now significant (although quite dependent on a single participant, which we stress as well). Regarding the genotype-phenotype associations tables, we now more clearly indicate what the contents correspond to. We also expanded the captions and no longer refer to Table 1. ________________________________________________________________________________________ *Q 1.5 A more fruitful discussion would be one that integrates findings with those from previous GWAS or microbiome studies. A more critical examination is necessary to determine the impact of averaging temporal data on genetic associations. The potential influence of environmental or behavioral factors on vaginal microbiota diversity warrants further investigation. The recommendations for future research are somewhat generic; more specific recommendations would improve impact.*

 Reply: We expanded on the discussion about the specificity of working with microbiota as a response trait in the introduction and, more thoroughly, in the discussion. About the effect of environmental/behavioural covariates, this is the case for most traits included in GWAS. The vaginal microbiota is actually interesting as a study subject because these covariates are well characterised (largely thanks to the high level of clustering of the microbiota), which makes it easy to control for them. ________________________________________________________________________________________ *Q 1.6 It has been noted that certain sections of the text contain dense jargon, which may be more effectively mitigated through the implementation of simpler language in certain sections. Minor typographical errors and inconsistencies in terminology (e.g., reference to CST4 vs. CST-4) occur on occasion. The inclusion of more informative legends and clearer labeling would enhance the comprehensibility of the figures. It is recommended that the formatting of select tables be revised to enhance clarity, particularly those that present cohort characteristics.*

 Reply: We went through the manuscript to try and remove the jargon as much as possible, to homogenise the notations, and clarify the captions. In particular, we mention as often as possible that CST-4 is lactobacillus-poor to facilitate the reading.
